# Risk of Healthcare-Associated *Clostridioides difficile* Infection During Pandemic Preparation: A Retrospective Cohort Study

**DOI:** 10.1016/j.gastha.2021.08.005

**Published:** 2022-02-03

**Authors:** L. Suarez, J. Kim, D.E. Freedberg, B. Lebwohl

**Affiliations:** 1 Vagelos College of Physicians and Surgeons, Columbia University, New York, New York; 2 Department of Medicine, Columbia University Irving Medical Center, New York, New York

*Clostridioides difficile* infection (CDI) is the most common cause of healthcare-associated infectious diarrhea. *C. difficile* spores are not killed by alcohol-based hand sanitizers, requiring healthcare workers to wash their hands thoroughly with soap and water to prevent transmission.^1^^,^^2^

Handwashing is a key intervention in reducing CDI, yet adherence to proper technique amongst healthcare workers remains variable and often substandard.^2^, ^3^, ^4^

In late 2019, novel coronavirus SARS-CoV-2 was identified, with the first COVID-19 case in the United States reported in January, 2020. Handwashing was emphasized to prevent spread of infection, including media coverage educating the public on proper technique. Therefore, we hypothesized that healthcare-associated CDI rates would decrease preceding the arrival of recognized community circulation of COVID-19 related to improved hand hygiene.

We performed a retrospective cohort study comparing the incidence and risk factors of healthcare-associated CDI from January 1–March 31, 2020 (prepandemic, when we anticipated improved handwashing) to January 1–March 31, 2019 (control). We similarly analyzed healthcare-associated non-*C. difficile* enteric infections (non-CDI). See Supplemental Text for detailed Methods.

We identified 13,336 hospital admissions (6447 in January–March, 2019; 6889 in January–March, 2020). Patients in 2020 were less likely to have hypoalbuminemia (30.9% vs 40.6%, *P* <.001) and vancomycin-resistant enterococci (VRE) isolation status (0.4% vs 1.3%, *P* <.001) but more likely to have a Charlson Comorbidity Index (CCI) ≥3 (59.6% vs 56.4%, *P* <.001) and receive antibiotics (65.0% vs 60.2%, *P* <.001) and high-risk antibiotics (45.3% vs 43.3%, *P* = .019).^5^

The overall positivity rate for *C. difficile* (measure of testing rate) was similar between the periods (12.8% vs 13.1%, *P* = .893). CDI occurred in 0.4% of admissions both years (*P* = .703, Table) with increased incidence in patients with ICU admission (*P* = .005), hypoalbuminemia (*P* = .014), methicillin-resistant *Staphylococcus aureus* isolation status (*P* = .037), VRE isolation status (*P* = .001), antibiotics (*P* <.001), and high-risk antibiotics (*P* = .004). Certain comorbidities were associated with CDI, including active leukemia (*P* <.001). The median hospitalization length was 19.3 vs 5.7 days for patients who did and did not develop CDI (*P* <.001).

**Table:** Univariable Analysis of Factors Associated With Developing *C. difficile* Infection During Hospitalization

Characteristic	*C. difficile* positive, n = 55 (0.4%)	Not *C. difficile* positive, n = 13,281 (99.6%)	*P* value
	n (%)	n (%)	
Time period			.703
2019	28 (0.4)	6419 (99.6)	
2020	27 (0.4)	6862 (99.6)	
Female sex	32 (0.4)	7348 (99.6)	.671
Age, median (IQR)	64 (52–75)	62 (42–75)	.311
Age, categorical			.159
18–39 y	8 (0.3)	3033 (99.7)	
40–59 y	11 (0.4)	3000 (99.6)	
60–75 y	24 (0.6)	4020 (99.4)	
>75 y	12 (0.4)	3228 (99.6)	
Race			.423
White	22 (0.5)	4553 (99.5)	
Black	12 (0.5)	2483 (99.5)	
Other/unknown	21 (0.3)	6245 (99.7)	
Ethnicity			.772
Hispanic	17 (0.4)	3869 (99.6)	
Not Hispanic/unknown	38 (0.4)	9412 (99.6)	
Admission service			.057
Medicine	39 (0.5)	8142 (99.5)	
Surgery	12 (0.5)	2397 (99.5)	
Neurology	1 (0.2)	474 (99.8)	
Obstetrics/gynecology	0 (0.0)	1729 (100.0)	
Other	3 (0.6)	539 (99.4)	
ICU admission	18 (0.7)	2417 (99.3)	.005
ICU type, n = 2399			.434
Allen	1 (0.3)	385 (99.7)	
Medical	4 (0.9)	423 (99.1)	
Cardiac	6 (1.4)	416 (98.6)	
Surgical	2 (0.6)	332 (99.4)	
Cardiothoracic	4 (0.8)	519 (99.2)	
Neurological	1 (0.3)	306 (99.7)	
ICU admission ≥24 h	18 (0.8)	2200 (99.2)	.001
ICU ≥24 h type, n = 2183			.604
Allen	1 (0.3)	335 (99.7)	
Medical	4 (1.0)	396 (99.0)	
Cardiac	6 (1.5)	402 (98.5)	
Surgical	2 (0.7)	304 (99.3)	
Cardiothoracic	4 (0.9)	460 (99.1)	
Neurological	1 (0.4)	268 (99.6)	
MRSA isolation status	3 (1.3)	232 (98.7)	.037
VRE isolation status	4 (3.5)	111 (96.5)	.001
Creatinine, median (IQR)	1.1 (0.8–2.0)	1.0 (0.7–1.5)	.090
Creatinine, categorical, n = 11,866			.031
≤ 1.5 mg/dL	35 (0.4)	8987 (99.6)	
>1.5 mg/dL	20 (0.7)	2824 (99.3)	
Albumin, median (IQR)	3.3 (2.8–3.9)	3.6 (3.1–4.0)	.009
Albumin, categorical, n = 9048			.014
< 3.4 g/dL	25 (0.8)	3165 (99.2)	
≥ 3.4 g/dL	23 (0.4)	5834 (99.6)	
Receipt of antibiotics during admission (excluding metronidazole and vancomycin)	50 (0.6)	8310 (99.4)	<.001
Receipt of high-risk antibiotics during admission^a^	35 (0.6)	5879 (99.4)	.004
Receipt of non–high-risk antibiotics (only) during admission	15 (0.6)	2431 (99.4)	.086
Comorbidities			
AIDS	1 (0.3)	321 (99.7)	1.000
Solid tumor, local	17 (0.6)	2650 (99.4)	.043
Solid tumor, metastatic	2 (0.4)	475 (99.6)	1.000
Leukemia	6 (2.6)	228 (97.4)	<.001
Lymphoma	1 (0.4)	280 (99.6)	1.000
Cerebrovascular accident or TIA	9 (0.6)	1434 (99.4)	.185
Congestive heart failure	11 (0.5)	2380 (99.5)	.688
Chronic kidney disease, moderate to severe	14 (0.8)	1742 (99.2)	.007
COPD	5 (0.4)	1309 (99.6)	.849
Connective tissue disease	5 (1.8)	280 (98.2)	.006
Dementia	3 (0.3)	863 (99.7)	1.000
Diabetes mellitus, uncomplicated	15 (0.5)	3215 (99.5)	.596
Diabetes mellitus, end-organ damage	14 (1.0)	1361 (99.0)	<.001
Hemiplegia	1 (0.7)	144 (99.3)	.453
Liver disease, mild	0 (0.0)	697 (100.0)	.118
Liver disease, moderate to severe	0 (0.0)	181 (100.0)	1.000
Myocardial infarction	7 (0.7)	930 (99.3)	.107
Peptic ulcer disease	1 (0.3)	291 (99.7)	1.000
Peripheral vascular disease	9 (1.1)	784 (98.9)	.005
Charlson Comorbidity Index			.095
0–2	17 (0.3)	5582 (99.7)	
3+	38 (0.5)	7699 (99.5)	
Duration of hospitalization, median (IQR)	19.3 (10.5–35.0)	5.7 (3.9–9.2)	<.001
Duration of hospitalization, categorical			<.001
<5 d	0 (0.0)	5581 (100.0)	
5–10 d	11 (0.2)	4716 (99.8)	
>10 d	44 (1.5)	2984 (98.5)	

AIDS, acquired immunodeficiency syndrome; COPD, chronic obstructive pulmonary disease; ICU, intensive care unit; IQR, interquartile range; MRSA, methicillin-resistant *Staphylococcus aureus*; TIA, transient ischemic attack; VRE, vancomycin-resistant enterococci.

a High-risk antibiotics were defined to include cephalosporins, monobactams, carbapenems, quinolones, and clindamycin.^5^

There was no association between year and CDI (odds ratio [OR] = 1.0; 95% confidence interval [CI] = 0.6–1.8). After adjusting for age, sex, and variables with *P* <.05, leukemia (OR = 3.6; 95% CI = 1.4–9.4), connective tissue disease (OR = 3.7; 95% CI = 1.4–10.0), and hospitalization >10 days (OR = 5.4; 95% CI = 2.5–11.6) were independent predictors of CDI.

Incidence of non-CDI was 0.6% and 0.5% in the 2019 and 2020 periods, respectively (*P* = .604). Intensive care unit (ICU) admission (*P* = .024), VRE isolation status (*P* = .001), high-risk antibiotics (*P* = .004), and certain comorbidities (but not overall CCI) were associated with infection. The median hospitalization length was 13.3 vs 5.7 days for patients who did and did not develop infection (*P* <.001).

There was no association between year and non-CDI (OR = 0.9; 95% CI = 0.6–1.5). After adjusting for age, sex, and variables with *P* <.05, independent predictors included VRE isolation status (OR = 4.2; 95% CI = 1.7–10.5) and hospitalization 6–10 days (OR = 3.3; 95% CI = 1.4–8.2) and >10 days (OR = 8.4; 95% CI = 3.5–20.3).

The rate of composite outcome was 0.9% in both periods (*P* = .856), and there was no association with year on multivariable analysis.

When we excluded patients admitted on or after March 3 (date of the first known COVID-19 admission at the medical center, 2020), the rate of CDI was 0.5% and 0.4% in 2019 and 2020 periods, respectively (*P* = .658). There was no difference in the rates of non-CDI or composite outcome between the periods. On multivariable analysis, there was no association between period and any of the outcomes.

We found no difference in the incidence of healthcare-associated CDI and non-CDI between January–March of 2019 and 2020, despite our hypothesis that there would be a decrease in 2020 related to improved hand hygiene.

Several studies have investigated CDI in the era of COVID-19. A study in Madrid, Spain, analyzed the incidence density of healthcare facility–associated CDI during the maximum incidence of COVID-19 compared with the same period in 2019 and found a nearly 70% decrease.^6^ A key difference between our studies is the period investigated, suggesting increased handwashing may have taken longer to take effect than we anticipated. A study at Mount Sinai Hospital (New York) hypothesized that CDI incidence may have increased during the pandemic owing to increased antibiotics.^7^ However, no significant difference in CDI was identified, despite a trend toward increased high-risk antibiotics in the COVID-19 period.

Both studies analyzed peak COVID-19 periods, subjecting them to additional confounders we attempted to avoid by studying the prepandemic period, such as hospital crowding and differences in patients admitted caused by risk factors for more severe COVID-19, requiring hospitalization.

A limitation of this study is that our hypothesis assumed handwashing improved in 2020, but we did not measure performance. Therefore, it is possible that there was no significant change in hand hygiene. A hospital in Jerusalem, Israel, investigated handwashing in the setting of COVID-19 and found that average compliance increased from 46% (January 2020) to 89% (April 2020).^8^ A study in the United States found that average performance increased from 46% (early January 2020) to 64% (late March 2020).^9^ Additionally, in a survey of 6463 US adults (March 19–April 9, 2020), 93% said they were “washing hands often with soap and water” to prevent coronavirus; however, the survey did not assess actual performance.^10^

This study is also limited by investigating a single hospital system (although it included two hospitals). The electronic medical record system changed in February 2020, which may have affected documentation/reporting of certain variables such as comorbidities—possibly explaining the increase in certain comorbidities and CCI in 2020.

This study also had a number of strengths, including the large number of admissions analyzed (n = 13,336) and identification of known risk factors for CDI, including antibiotics and ICU admission. By analyzing the months preceding the peak of COVID-19, we avoided additional differences between the periods.

Future studies may investigate long-term changes in handwashing and infection rates. Additionally, future studies may investigate the incidence of these infections during peak pandemic in a hospital that had few COVID-19 cases, where there was likely still emphasis placed on handwashing to prevent transmission.
