# Supplementary material for: Risk of Healthcare-Associated Clostridioides difficile Infection During Pandemic Preparation: A Retrospective Cohort Study
Source: Gastro Hep Adv. 2022 Feb 3;1(1):8–11. doi: 10.1016/j.gastha.2021.08.005 (PMC8818443; doi:10.1016/j.gastha.2021.08.005)
Supplement: Supplemental Methods [file mmc1.docx]

**Methods**

*Study design*

We performed a retrospective cohort study comparing the incidence of healthcare-associated CDI from January 1 – March 31, 2020 (pre-pandemic period, when we anticipated improved hand hygiene) to the incidence of healthcare-associated CDI from January 1 – March 31, 2019 (control period). We conducted a similar analysis on the incidence of non-*C. difficile* enteric infections (non-CDI), using stool gastrointestinal pathogen PCR (GIPCR), which may also have decreased with improved hand hygiene. We chose to analyze the pre-pandemic period rather than peak-pandemic period to avoid potential confounders between the two periods, such as hospital crowding, differences in the patients admitted due to risk factors for severe COVID-19, or even the effect of COVID-19 itself on CDI.

*Population*

Using our hospital’s electronic medical record, we identified patients admitted to NewYork-Presbyterian/Columbia University Irving Medical Center and The Allen Hospital from January 1 – March 31 of 2019 and 2020. Patients ≥18 years were included in the study if they were admitted from the emergency department, through direct admission from home, or after ambulatory surgery and had a hospital stay ≥72 hours. The study protocol was approved by the institutional review board of Columbia University Irving Medical Center.

*Exposure and outcome definitions*

The primary exposure was the year of admission for each patient (i.e., pre-pandemic period of January – March, 2020 versus January – March, 2019). Hospital-associated CDI was defined as a positive polymerase chain reaction (PCR) test for the *C. difficile* toxin B gene from an unformed stool sample ≥72 hours after presentation (defined as time of triage vitals for patients who presented to the emergency department), followed by the receipt of anti-CDI antibiotics. Hospital-associated non-CDI was defined as a positive stool GIPCR test ≥72 hours after presentation. Patients were followed until discharge or April 30 for the development of the outcome.

*Covariates*

Automated electronic queries were used to acquire and examine the following potential risk factors for CDI and non-CDI that may have differed by year: demographics, admission service, intensive care unit (ICU) status, ICU type, isolation status (methicillin-resistant *Staphylococcus aureus* [MRSA] and vancomycin-resistant enterococci [VRE]), laboratory values on admission (creatinine and albumin), receipt of antibiotics during admission (separated into high-risk and non-high-risk for development of CDI), various comorbidities (using diagnosis codes associated with each patient’s medical record), Charlson Comorbidity Index (CCI), and duration of hospitalization (date of admission to date of discharge). We defined high-risk antibiotics to include cephalosporins, monobactams, carbapenems, quinolones, and clindamycin [5]. When assessing ICU status as a covariate, we separately analyzed any ICU stay and ICU stays of ≥24 hours, as shorter stays may have been due to routine postoperative care rather than severe illness.

*Statistical analysis*

Continuous variables were expressed as medians and interquartile ranges (IQR). Categorical variables were summarized as counts and percentages. Chi-square or Fisher’s exact tests were used to compare categorical variables. Mann-Whitney U test or Student’s t test was used to compare non-normally distributed continuous variables. A multivariable logistic regression model was built to examine the relationship between admission period and infection status (CDI, non-CDI, or either infection) after adjusting for other variables, using odds ratios (OR) and confidence intervals (CI). Variables with a p value >0.05 on univariable analysis were removed, except for age and sex, which we retained in the model, *a priori*. High-risk and non-high-risk antibiotic categories were excluded from the model due to collinearity. Individual comorbidities were included in the model instead of CCI score due to collinearity between individual comorbidities and overall score, and due to strong associations between some individual comorbidities and outcomes on univariable analysis. Statistical calculations were performed in STATA 16 (Stata Corp, College Station, TX), and a two-sided p value <0.05 was considered statistically significant.

*Sensitivity analysis*

To test whether the relationship between year of admission and CDI was confounded by the influx of COVID-19 cases, a sensitivity analysis was performed, excluding patients admitted on or after March 3 of each year —the date of the first known COVID-19 admission in 2020 at the medical center. Since the medical center changed its electronic health record vendor from Allscripts to Epic in February 2020, a random selection of 50 *C. difficile* and GIPCR results were manually checked to ensure the validity of these data in both systems.
